# Targeting CARD6 attenuates spinal cord injury (SCI) in mice through inhibiting apoptosis, inflammation and oxidative stress associated ROS production

**DOI:** 10.18632/aging.102561

**Published:** 2019-12-16

**Authors:** Jiang Lin Wang, Xiao Luo, Li Liu

**Affiliations:** 1 Department of Pain Management, The Affiliated Hospital of Southwest Medical University, Luzhou 646000, Sichuan Province, China; 2 Department of Pain Management, The Third Xiangya Hospital of Central South University, Changsha 410013, Hunan Province, China; 3 Department of Anesthesiology, The Affiliated Hospital of Southwest Medical University, Luzhou 646000, Sichuan Province, China

**Keywords:** spinal cord injury (SCI), CARD6, apoptosis, inflammation, ROS

## Abstract

Spinal cord injury (SCI) causes long-term and severe disability, influencing the quality of life and triggering serious socioeconomic consequences. Lack of effective pharmacotherapies for SCI is largely attributable to an incomplete understanding of its pathogenesis. Caspase recruitment domain family member 6 (CARD6) was initially suggested to be a protein playing significant role in NF-κB activation. However, the effects of CARD6 on SCI progression remain unknown. In this study, the wild type (CARD6+/+), CARD6 knockout (CARD6-/-) and CARD6 transgenic (TG) mice were subjected to a SCI model in vivo, and in vitro experiments were conducted by treating microglia cells with lipopolysaccharide (LPS). Here, we identified CARD6 as a suppressor of SCI in mice. CARD6 knockout significantly accelerated functional deficits, neuron death and glia activation, whereas CARD6 overexpression resulted in the opposite effects. Both in vivo and in vitro SCI models suggested that CARD6 knockout markedly promoted apoptosis by increasing Cyto-c release to cytosol from mitochondria and activating Caspase-3 signaling. In addition, CARD6 knockout mice exhibited stronger inflammatory response after SCI, as evidenced by the significantly elevated expression of pro-inflammatory cytokines TNF-α, IL-1β and IL-6, which was largely through enhancing the activation of NF-κB signaling.

## INTRODUCTION

Spinal cord injury (SCI) results in long-term and severe disability, which influences the quality of life and induces serious socioeconomic consequences [1, 2]. SCI consists of two injuries, including the primary injuries that occur at initial impact and the secondary injuries that develop soon after the injury. Primary injuries involve mechanical compression of the spine. Secondary SCI effects, including posttraumatic inflammation, oxidative stress, motor neuron apoptosis and necrosis, results in further damage to the initial injury [3–5]. In addition, SCI is followed by an acute but long-lasting inflammatory response, marked by invasion of blood-borne cells and activation of endogenous cells and a significant increase in reactive oxygen species (ROS) generation [6]. ROS accumulation could enhance neuronal apoptosis via protein breakdown, lipid peroxidation and DNA damage [7, 8]. Oxidative stress leads to the activation of glial cells and promotes the release of pro-inflammatory factors [9]. In addition, apoptosis also plays a critical role in the secondary damage in animal models and in human tissue, resulting in gradual degeneration of the spinal cord [3, 10]. Therefore, exploring novel targets to develop effective treatments against ROS production, inflammation and apoptosis could improve SCI, contributing to the functional recovery following SCI.

The caspase recruitment domain (CARD) is a homotypic protein-protein interaction module that links components of signal transduction pathways involved in the modulation of apoptosis or innate immunity [11, 12]. CARD6, as a member of CARD family, is initially suggested to activate NF-κB signaling by several independent pathways [13]. CARD6 could also interact with RIP2 (also known as RICK or CARDIAK), a CARD-containing member of the RIP family of protein kinases to mediate NF-κB activation [12, 14]. Neoexpression of CARD6 might be associated with NF-κB activation in the cancers and plays a potential role in the development of many types of gastrointestinal cancers [15]. However, other studies suggested that CARD6 inhibited NF-κB activation by NOD1 or RIPK2 [16]. In addition, CARD6-deficient mice did not show abnormalities in signaling pathways that regulate the innate and adaptive immune responses [14]. Also, pressure overload-increased CARD6 protected against cardiac hypertrophy [17]. Recently, CARD6 was suggested to reduce liver damage, alleviate cell death, and prevent inflammation in hepatic ischemia/reperfusion (I/R) injury [18]. Therefore, the pathophysiological function of CARD6 is not completely understood. Given the significant role of CARD6 in regulating inflammation and apoptosis, we supposed that the expression change of CARD6 might be associated with the development of SCI.

In the present study, for the first time we found that CARD6 expression was gradually down-regulated in spinal cord tissues of SCI mice, indicating its potential role during SCI development. CARD6^-/-^ mice after SCI showed significantly extensive neuronal death, glial activation, inflammation and oxidative stress, along with severer functional deficits compared to CARD6^+/+^ mice. However, CARD6 overexpression resulted in the opposite effects. Importantly, the *in vitro* studies demonstrated that CARD6 knockdown-enhanced cell death, inflammatory response and oxidative stress was largely dependent on ROS production. Collectively, our data revealed a previously unappreciated role for CARD6 in SCI pathogenesis and identified the CARD6 as a promising target in the treatment of SCI.

## RESULTS

### CARD6 expression is up-regulated in the spinal dorsal horn of mice

**Figure 1:**
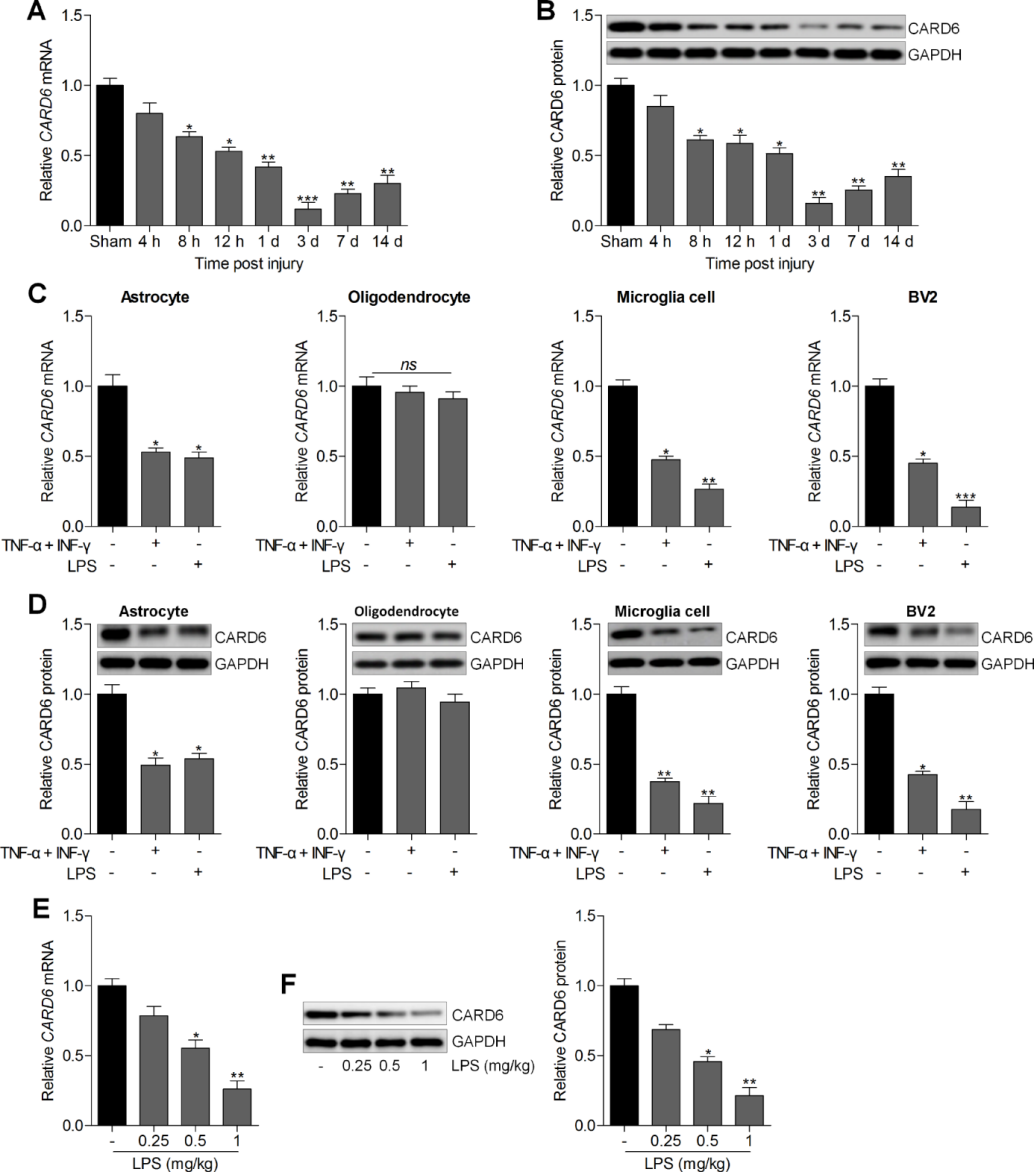
**CARD6 expression is up-regulated in the spinal dorsal horn of mice.** Representative (**A**) RT-qPCR and (**B**) western blot analysis of CARD6 expression in lumbar spinal tissues. ^*^p < 0.05 and ^**^p < 0.01 vs the Sham group. (**C**) The mRNA and (**D**) protein expression levels of CARD6 in primary cultured astrocytes, oligodendrocytes, microglia cells and mouse microglia BV2 cells incubated with TNF-α (10 ng/ml) plus IFN-γ (10 ng/ml) or LPS (100 ng/ml) for 24 h by RT-qPCR and western blot analysis, respectively. ^*^p < 0.05 and ^**^p < 0.01 vs the Con group in the absence of any treatments. (**E**) RT-qPCR and (**F**) western blot analysis of CARD6 expression in spinal cord tissues. Data represented means ± SEM (n=6 each group).

First, the expression change of CARD6 was calculated in spinal cord tissues of wild type mice with or without SCI. As shown in Figure 1A and 1B, the CARD6 mRNA and protein expression levels were reduced at different time points after SCI compared to the expression in the sham group. In addition, RT-qPCR and western blot analysis suggested that CARD6 expression levels were markedly reduced in primary astrocytes, microglia cells and mouse BV2 microglia cells induced by TNF-α/IFN-γ or LPS. However, no significant difference was observed in the expression change of CARD6 in primary cultured oligodendrocytes treated with TNF-α/IFN-γ or LPS compared to the Con group in the absence of any treatments (Figure 1C and 1D). To confirm this LPS-induced BV2 cell model really mimic SCI *in vivo*, the expression of CARD6 was measured in spinal cord after injection of LPS *in vivo*. RT-qPCR and western blot analysis demonstrated that the CARD6 mRNA and protein expression levels in spinal cord segments (dorsal part of L4-L5) were significantly reduced by LPS in a dose-dependent manner (Figure 1E and 1F). These results demonstrated that the expression change of CARD6 might be involved in the progression of SCI.

### CARD6 knockout accelerates SCI in mice

**Figure 2:**
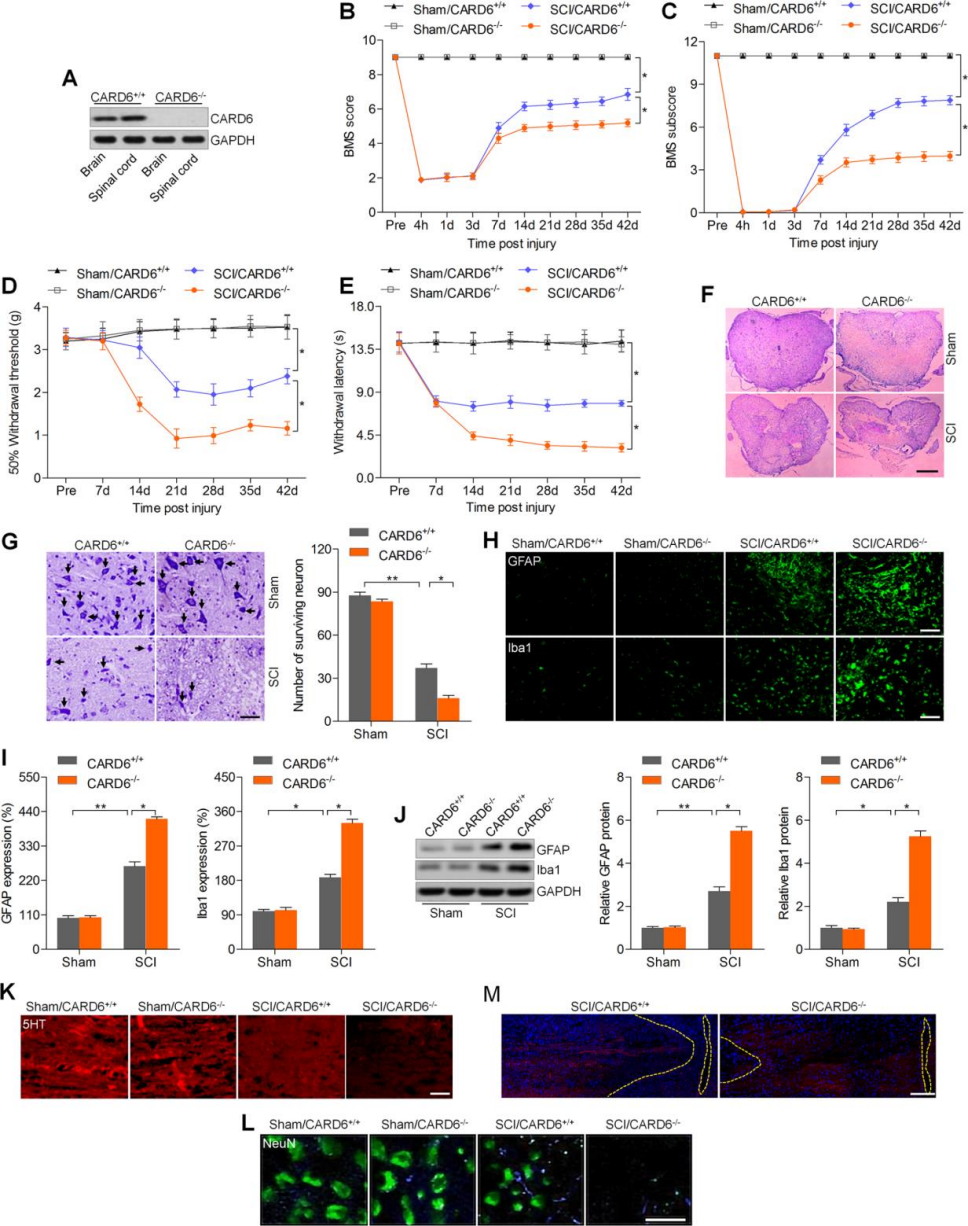
**CARD6 knockout accelerates SCI in mice.** (**A**) Western blot analysis CARD6 protein expression in brain and lumbar spinal tissues from CARD6^+/+^ or CARD6^-/-^ mice. (**B**) The BMS scores and (**C**) BMS subscores were measured in each group of mice. (**D**) The withdrawal threshold was measured to calculate the mechanical hypersensitivity in each group of mice. (**E**) The withdrawal latency was measured to determine the thermal hypersensitivity in each group of mice. (**F**) H&E staining of adjacent sections. Scale bar: 100 μm. (**G**) Nissl staining of lumbar spinal cords in the ventral horn of gray matter from mice 3 days after SCI. The number of survived neuron was quantified. Scale bar: 100 μm (black arrows: the normal surviving neurons). (**H**) IF staining of GFAP and Iba-1 in the spinal dorsal horn of mice. Scale bar: 100 μm. (**I**) Quantification of GFAP and Iba-1 following IF analysis. (**J**) Western blot analysis of GFAP and Iba-1 in the lumbar spinal cord segments. (**K**, **L**) 5-HT and NeuN staining of axons in white matter tracts at the site of cord compression. (**M**) BDA-labeled the corticospinal tracts in proximity of the lesion sites of CARD6^+/+^ or CARD6^-/-^ mice after SCI for 3 days. Data represented means ± SEM (n=8 each group). ^*^p < 0.05 and ^**^p < 0.01.

To reveal the physiological function of CARD6 in SCI, CARD6 knockout mice were used. CARD6 expression was undetectable in brain and lumbar spinal tissues from CARD6^-/-^ mice (Figure 2A). Then, the functional role of CARD6 was investigated in mice 3 days after SCI across our study. Mice from sham groups showed no locomotor impairment and maintained full marks in the BMS score and subscore for 42 days. SCI mice exhibited lower BMS score and subscore for 42 days, which were further reduced in CARD6^-/-^ mice following SCI (Figure 2B and 2C). The withdrawal thresholds in the SCI mice were lower than in those in the Sham mice from days 14 to 42 after SCI, which was further accelerated in mice with the loss of CARD6, demonstrating severer mechanical hypersensitivity (Figure 2D). SCI-induced thermal hypersensitivity was markedly aggravated in CARD6^-/-^ mice, as evidenced by the further reduced withdrawal latency (Figure 2E). H&E staining indicated that the dorsal white matter and central gray matter showed obvious damages in the SCI/CARD6^+/+^ group relative to that in the sham group. Severer injury was observed in CARD6^-/-^ mice following SCI, which was comparable to the SCI/CARD6^+/+^ group (Figure 2F). Nissl staining indicated that SCI mice had numerous necrotic neurons, and this process was significantly accelerated in CARD6^-/-^ mice after SCI (Figure 2G). Following SCI, the expression levels of GFAP and Iba-1, representing the activation of astrocytes and microglia cells, respectively, were further promoted by CARD6 ablation by IF staining and western blot analysis (Figure 2H–2J). To further calculate neuronal damage, axons of the descending raphespinal tract were explored in the spinal cord. At the center of the compression, a significant loss 5HT-positive axons and NeuN expression occurred compared to the Sham group, while being further accelerated in CARD6^-/-^ mice, as proved by the weaker fluorescence (Figure 2K and 2L). Additionally, the corticospinal tracts sprouting and retraction bulb indexes at the spinal cord level were then measured. The distances of corticospinal tracts to the lesion site elevated in CARD6^-/-^ mice after SCI for 3 days when compared with those in CARD6^+/+^ mice, which demonstrated an elevated dieback of the corticospinal tracts (Figure 2M). These observations demonstrated that CARD6 played a critical role in SCI development.

### CARD6 knockout enhances apoptosis after SCI

**Figure 3:**
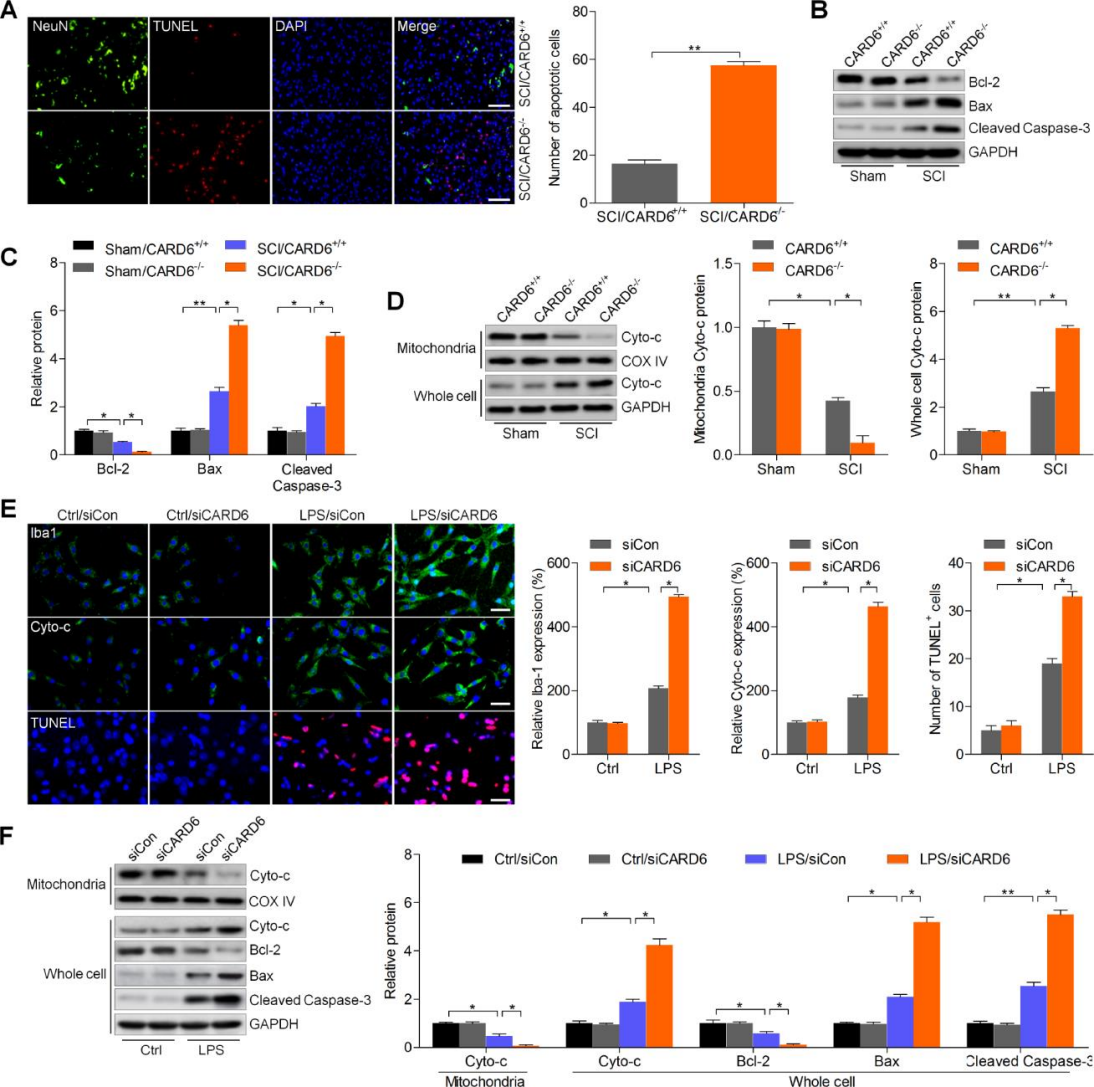
**CARD6 knockout enhances apoptosis after SCI.** (**A**) Representative images of NeuN/TUNEL double staining in dorsal horn of mice. The number of apoptotic cells (TUNEL positive) was quantified. Scale bar: 100 μm. (**B**, **C**) Western blot analysis of Bcl-2, Bax and cleaved Caspase-3 protein expression levels in the lumbar spinal cord segments. (**D**) Western blot analysis of mitochondrial and whole cell Cyto-c in the lumbar spinal cord segments. (**E**, **F**) BV2 cells with or without CARD6 knockdown were treated with LPS (100 ng/ml) for 24 h. (**E**) Then, IF staining was used to determine Iba1, Cyto-c and TUNEL levels. The quantification of Iba1, Cyto-c and TUNEL expression levels was exhibited. Scale bar: 50 μm. (**F**) Western blot analysis was used to calculate Cyto-c protein levels in mitochondria, or Cyto-c, Bcl-2, Bax and cleaved Caspase-3 expression in whole cell as indicated. Data represented means ± SEM (n=8 each group for *in vivo* studies; n=6 each group for *in vitro* studies). ^*^p < 0.05 and ^**^p < 0.01.

In order to explore if CARD6 could modulate apoptosis induced by SCI, TUNEL staining was performed. As shown in Figure 3A, SCI-triggered apoptosis was significantly worsened by CARD6 knockout in dorsal horn of mice, as evidenced by the increased number of TUNEL-positive cells. Consistently, the anti-apoptotic protein Bcl-2 was decreased, and the pro-apoptotic molecule Bax was increased in lumbar spinal cord segments of CARD6^-/-^ mice following SCI, which were comparable to the SCI/CARD6^+/+^. Moreover, the markedly elevated expression of cleaved Caspase-3 was detected in lumbar spinal cord segments of CARD6^-/-^ mice after SCI (Figure 3B and 3C). In addition, CARD6^+/+^ mice with SCI showed significantly up-regulated release of Cyto-c into the cytoplasm from the mitochondria, and this apoptotic effect was, however, aggravated in CARD6^-/-^ mice (Figure 3D). Then, the effects of CARD6 on apoptosis in SCI were further investigated using BV2 cells transfected with siCARD6 or siCon. As shown in Supplementary Figure 1A and 1B, CARD6 expression was effectively reduced by the transfection with siCARD6. IF staining in Figure 3E indicated that BV2 cells with CARD6 knockdown showed stronger intensity and expression of Iba-1 after LPS stimulation, indicating greater activation of BV2 cells. Stronger immunoreactivity of cytosolic Cyto-c and higher levels of TUNEL-positive cells were observed in CARD6 knockdown BV2 cells following LPS exposure, which were comparable to the LPS/siCon group. Finally, LPS-induced release of Cyto-c from mitochondria to cytoplasm was markedly accelerated in CARD6 knockdown cells, accompanied with higher expression of Bax and cleaved Caspase-3. In contrast, the expression of pro-survival protein Bcl-2 inhibited by LPS was further down-regulated by CARD6 suppression in LPS-stimulated BV2 cells (Figure 3F). These findings indicated that suppressing CARD6 expression could promote SCI development partly by exacerbating apoptosis.

### CARD6 knockout accelerates inflammatory response in mice after SCI

**Figure 4:**
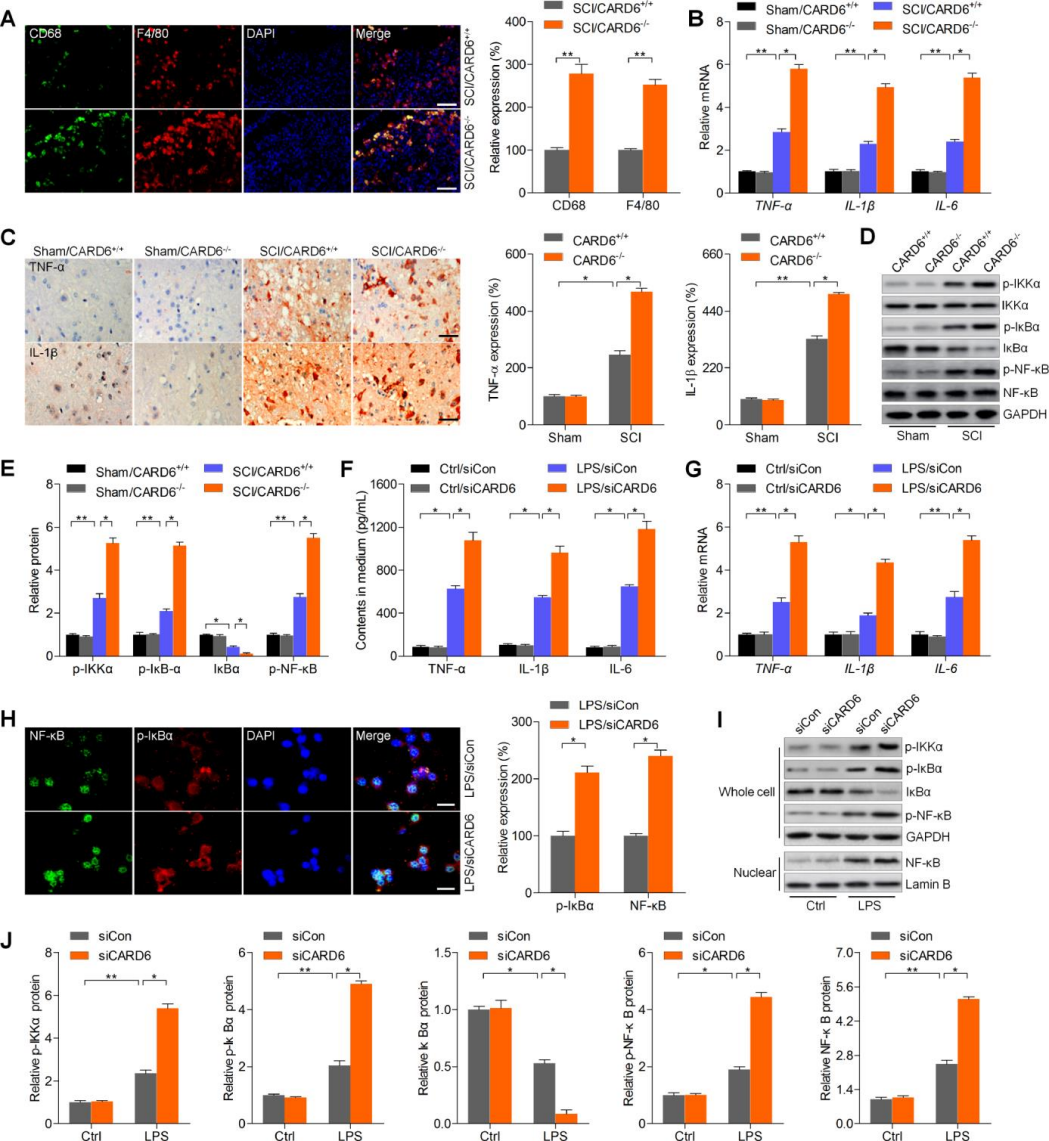
**CARD6 knockout accelerates inflammatory response in mice after SCI.** (**A**) Representative images of CD68/F4/80 double staining by IF in dorsal horn of mice. The relative expression of CD68 and F4/80 was quantified. Scale bar: 100 μm. (**B**) RT-qPCR analysis of TNF-α, IL-1β and IL-6 mRNA levels in the lumbar spinal cord segments. (**C**) Representative images of TNF-α and IL-1β and by IHC staining in dorsal horn of mice. The relative expression of TNF-α and IL-1β was quantified. Scale bar: 100 μm. (**D**, **E**) Western blot analysis of p-IKKα, p-IκBα, IκBα and p-NF-κB protein expression levels in the lumbar spinal cord segments. (**F**–**J**) BV2 cells were transfected with siCARD6 or siCon for 24 h, followed by LPS exposure for another 24 h. Then, all cells were collected for further studies. (**F**) TNF-α, IL-1β and IL-6 contents in medium were assessed by ELISA. (**G**) TNF-α, IL-1β and IL-6 mRNA levels in cells were measured using RT-qPCR analysis. (**H**) Representative images of p-IκBα and NF-κB double staining by IF in cells. The quantification of p-IκBα and NF-κB expression levelv was exhibited. Scale bar: 50 μm. (**I**, **J**) Protein expression levels of p-IKKα, p-IκBα, IκBα and p-NF-κB in whole cells, and NF-κB in nuclear were determined by western blot analysis. Data represented means ± SEM (n=8 each group for *in vivo* studies; n=6 each group for *in vitro* studies). ^*^p < 0.05 and ^**^p < 0.01.

Excessive inflammation is involved in SCI progression, and CARD6 was previously suggested to modulate inflammatory response [18, 19]. Thus, we subsequently attempted to explore if CARD6 could modulate inflammation to regulate SCI development. IF staining suggested that the expression of macrophage markers CD68 and F4/80, playing crucial role in eliciting inflammation, was markedly intensified in CARD6^-/-^ mice after SCI, which was comparable to the SCI/CARD6^+/+^ group of mice (Figure 4A). Then, RT-qPCR and/or IHC analysis indicated that SCI-induced increase of pro-inflammatory cytokines TNF-α, IL-1β and IL-6 was further promoted by CARD6 knockout compared with those of CARD6^+/+^ mice after SCI (Figure 4B and 4C). The stimulation of NF-κB, a pivotal modulator of inflammation, was enhanced by CARD6 knockout after SCI, also as evidenced by the increased expression of phosphorylated IKKα, IκBα, and reduced IκBα (Figure 4D and 4E). Moreover, LPS-stimulated release or expression of TNF-α, IL-1β and IL-6 in medium or BV2 cells was further elevated by siCARD6 (Figure 4F and 4G). As shown in Figure 4H, CARD6 knockdown markedly promoted the expression of nuclear NF-κB and p-IκBα compared to siCon group of BV2 cells with LPS stimulation. Moreover, LPS-induced up-regulation of p-IKKα, p-IκBα, and p-NF-κB in whole cell was further elevated by CARD6 knockdown, which was along with a significant increase in nuclear NF-κB expression. However, an opposite expression change of total IκBα was observed in whole cell (Figure 4I and 4J). Collectively, the results above indicated that CARD6-regulated SCI was at least partly associated with inflammatory response through NF-κB signaling.

### CARD6 ablation promotes oxidative stress after SCI

**Figure 5:**
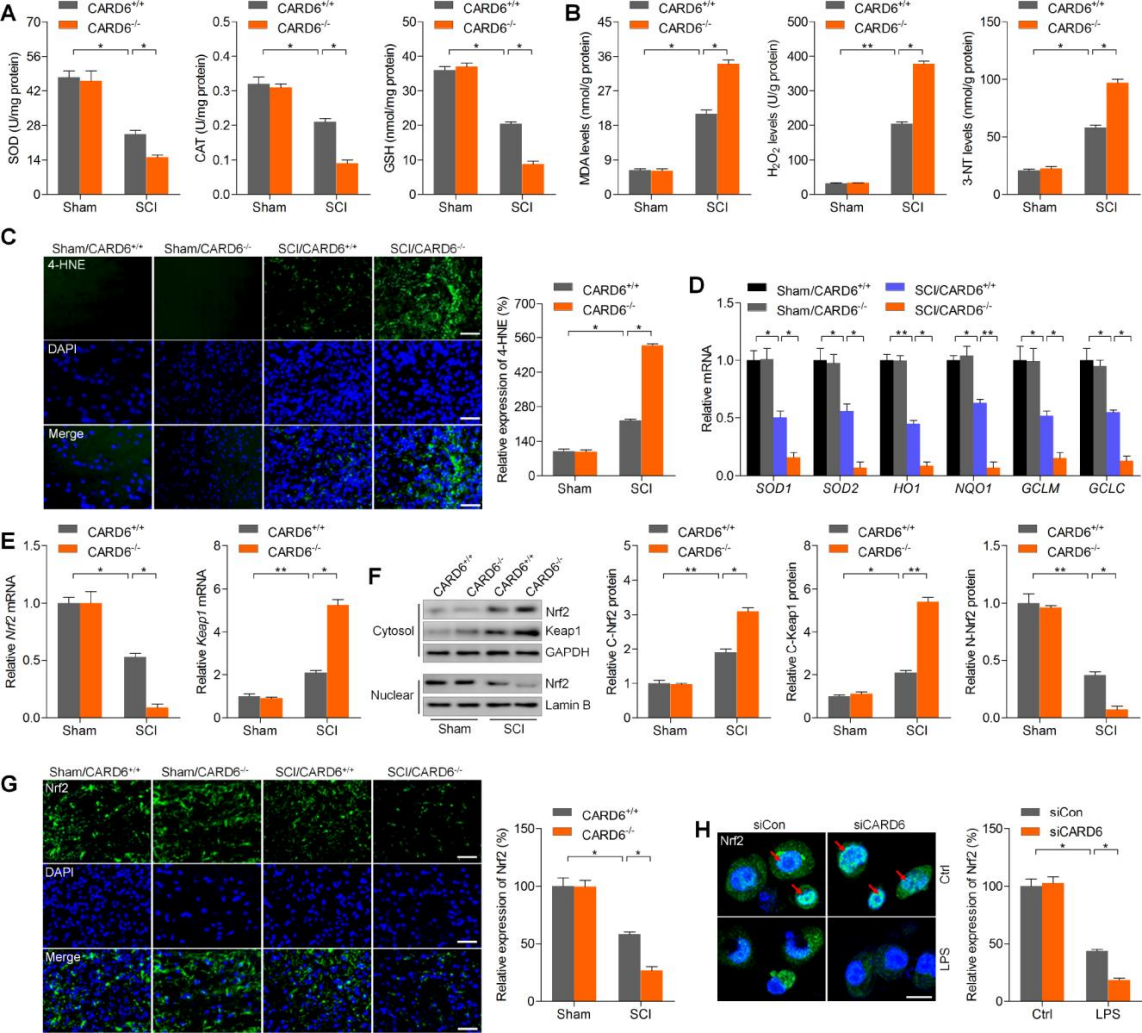
**CARD6 knockout promotes oxidative stress after SCI.** Measurements of (**A**) SOD, CAT, GSH, (**B**) MDA, H_2_O_2_ and 3-NT in spinal cords of mice at 3 days after SCI. (**C**) Representative images of 4-HNE by IF staining in dorsal horn of mice. The relative expression of 4-HNE was quantified. Scale bar: 100 μm. (**D**) RT-qPCR analysis of SOD1, SOD2, HO1, NQO1, GCLM and GCLC mRNA levels in the lumbar spinal cord segments. (**E**) RT-qPCR analysis of Nrf2 and Keap1 mRNA expression levels in the lumbar spinal cord segments. (**F**) Western blot analysis of cytosolic Nrf2 and Keap1, and nuclear Nrf2 protein expression levels in the lumbar spinal cord segments. (**G**) Representative images of Nrf2 by IF staining in dorsal horn of mice. The quantification of Nrf2 expression levels was showed. Scale bar: 100 μm. (**H**) IF staining of Nrf2 in BV2 cells transfected with siCARD6 followed by 24 h of LPS (100 ng/ml) stimulation. Then, Nrf2 expression levels were quantified. Scale bar: 25 μm. Data represented means ± SEM (n=8 each group for *in vivo* studies; n=6 each group for *in vitro* studies). ^*^p < 0.05 and ^**^p < 0.01.

Oxidative stress leads to microglial and astrocyte activation, promoting the release of pro-inflammatory cytokines [20]. ROS accumulation is also important for apoptosis induction during SCI [21]. We further explored whether CARD6 could influence oxidative stress after SCI. Under sham condition, CARD6 showed no effects on basal oxidative stress. Nevertheless, CARD6^-/-^ markedly reduced the activities or levels of anti-oxidants SOD, CAT and GSH compared to those of CARD6^+/+^ mice after SCI (Figure 5A). In contrast, the levels of oxidative stress markers of damaged lipid (MDA) [22] and protein (3-NT) [23] in spinal cord tissues were markedly promoted in CARD6^-/-^ mice following SCI compared to those in CARD6^+/+^ mice (Figure 5B). Consistently, SCI-induced increase of 4-HNE, as a major by-product of lipid peroxidation, a process that is exacerbated under oxidative stress conditions, was further accelerated by CARD6 knockout in mice after SCI (Figure 5C). In order to calculate if Nrf2 anti-oxidant signaling pathway was involved in CARD6-regulated SCI, the mRNA expression levels of SOD1, SOD2, HO1, NQO1, GCLM and GCLC were measured. As shown in Figure 5D, SCI mice with CARD6 knockout showed remarkable decreases in the expression of SOD1, SOD2, HO1, NQO1, GCLM and GCLC compared with CARD6^+/+^ mice. Then, we found that Nrf2 mRNA levels were down-regulated in SCI mice, while Keap1 was up-regulated. Notably, stronger expression change of Nrf2 and Keap1 was detected in SCI mice with CARD6^-/-^ (Figure 5E). Moreover, immunoblotting analysis indicated that Nrf2 and Keap1 expression in cytosol induced by SCI was further accelerated in CARD6^-/-^ mice after SCI. However, nuclear Nrf2 expression decreased by SCI was further reduced by CARD6 knockout (Figure 5F). Evidently, CARD6 knockout-reduced Nrf2 expression was confirmed by IF staining in mice following SCI (Figure 5G). Also, in LPS-treated BV2 cells, CARD6 knockdown further decreased nuclear Nrf2 translocation, which was comparable to the LPS/siCon group (Figure 5H). Together, these results indicated that CARD6 could regulate oxidative stress to control SCI development through Nrf2 signaling.

### CARD6 knockdown-promoted oxidative stress is ROS dependent in LPS-incubated BV2 cells

**Figure 6:**
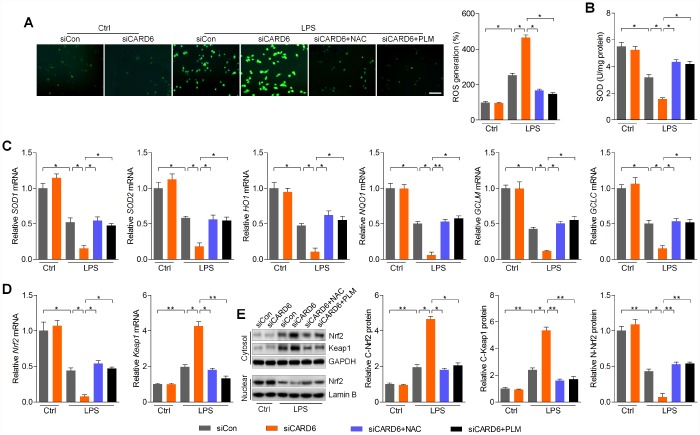
**CARD6 knockdown-promoted oxidative stress is ROS dependent in LPS-incubated BV2 cells.** (**A**–**E**) BV2 cells transfected with or without siCARD6 were pre-treated with NAC (5 mM) or PLM (10 μM) for 2 h, followed by LPS (100 ng/ml) treatment for another 24 h. Then, all cells were collected for further studies. (**A**) DCF-DA analysis was used for the calculation of ROS production. Scale bar: 100 μm. (**B**) SOD activity in cells was measured. (**C**) RT-qPCR analysis was used to measure the mRNA levels of SOD1, SOD2, HO1, NQO1, GCLM and GCLC in cells. (**D**) Nrf2 and Keap1 mRNA expression levels were determined by RT-qPCR. (**E**) Cytosolic Nrf2 and Keap1, and nuclear Nrf2 expression levels were assessed using western blot analysis. Data represented means ± SEM (n=6 each group). ^*^p < 0.05 and ^**^p < 0.01.

Considering the essential role of ROS production in regulating apoptosis and inflammation, we then inhibit ROS using NAC and PLM to further explore the effects of CARD6 on SCI [24]. As shown in Figure 6A, LPS-induced ROS generation was further promoted by CARD6 knockdown in BV2 cells. Intriguingly, CARD6 knockdown-promoted ROS accumulation was markedly abolished by NAC or PLM pre-treatment in LPS-incubated cells. In contrast, SOD activity suppressed by LPS was further worsened by CARD6 knockdown, and obviously this effect was apparently abrogated by NAC or PLM pre-treatment (Figure 6B). Furthermore, reducing CARD6 expression significantly decreased the mRNA levels of SOD1, SOD2, HO1, NQO1, GCLM and GCLC in LPS-stimulated BV2 cells, which was comparable to the LPS/siCon group. Notably, pre-treatment of NAC or PLM clearly diminished the effects of siCARD6 (Figure 6C). As expected, siCARD6 further decreased Nrf2 mRNA levels and increased Keap1 in LPS-exposed BV2 cells, while these effects were reversed by NAC or PLM (Figure 6D). Also, CARD6 knockdown cells showed higher Nrf2 and Keap1 expression in cytosol than that of the siCon cells with LPS exposure, which was diminished by NAC or PLM pre-treatment. In contrast, LPS-decreased expression of Nrf2 in nuclear was further down-regulated by CARD6 silence, which was, however, markedly rescued by NAC or PLM (Figure 6E). Taken together, these results suggested that CARD6-regulated oxidative stress in SCI might be ROS dependent.

### CARD6 silence-accelerated apoptosis and inflammation are regulated by ROS production in BV2 cells exposed to LPS

**Figure 7:**
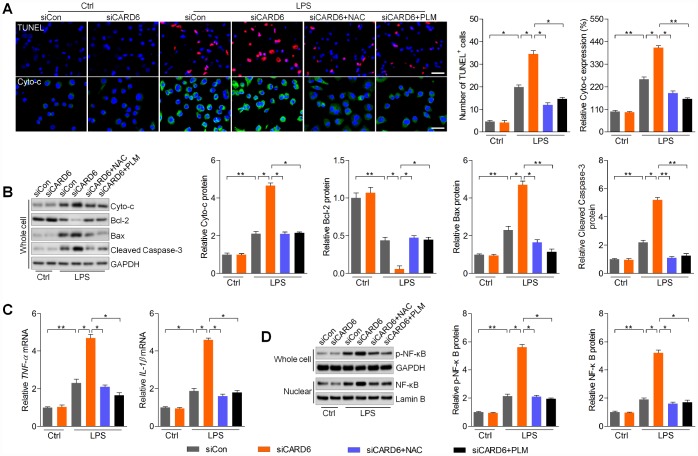
**CARD6 silence-accelerated apoptosis and inflammation are regulated by ROS production in BV2 cells exposed to LPS.** (**A**–**D**) BV2 cells in the presence or absence of siCARD6 transfection were pre-treated with NAC (5 mM) or PLM (10 μM) for 2 h, and then were subjected to LPS (100 ng/ml) exposure for another 24 h. Subsequently, all cells were harvested for further analysis. (**A**) TUNEL and Cyto-c staining of BV2 cells. TUNEL- and Cyto-c-positive cells were quantified. Scale bar: 100 μm. (**B**) Protein expression levels of Cyto-c, Bcl-2, Bax and cleaved Caspase-3 were evaluated by western blot analysis. (**C**) RT-qPCR analysis of TNF-α and IL-1β in cells. (**D**) Protein expression levels of phosphorylated NF-κB in whole cells and NF-κB in nuclear were measured by western blot analysis. Data represented means ± SEM (n=6 each group). ^*^p < 0.05 and ^**^p < 0.01.

In this regard, we found that siCARD6-promoted apoptosis was markedly alleviated by NAC or PLM pre-treatment in LPS-incubated BV2 cells, as evidenced by the reduced number of TUNEL-positive cells and expression of cytosolic Cyto-c (Figure 7A). Moreover, siCARD6-promoted expression of Cyto-c, Bax and cleaved Caspase-3 in LPS-stimulated BV2 cells was abolished by the pre-treatment of NAC or PLM, whereas Bcl-2 expression was improved (Figure 7B). Subsequently, we found that NAC or PLM treatment significantly down-regulated TNF-α and IL-1β mRNA levels elevated by siCARD6 in BV2 cells exposed to LPS (Figure 7C). Also, cellular p-NF-κB and nuclear NF-κB expression potentiated by siCARD6 were apparently abolished by NAC or PLM in LPS-treated BV2 cells (Figure 7D). Collectively, these results demonstrated that CARD6-regulated apoptosis and inflammation was dependent on ROS production to meditate the progression of SCI.

### CARD6 up-regulation alleviates SCI and improves functional recovery in mice

**Figure 8:**
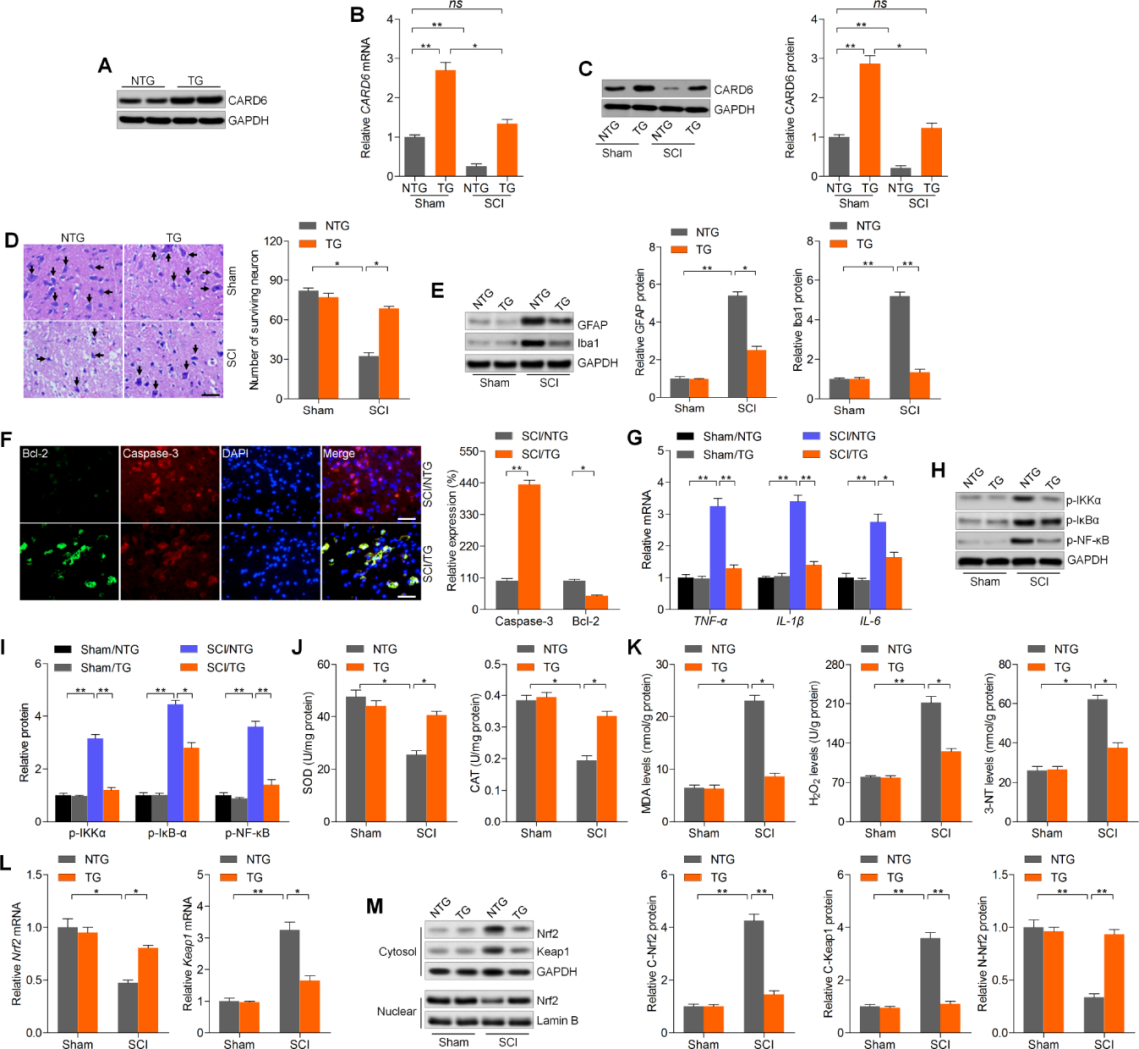
**CARD6 up-regulation alleviates SCI in mice.** (**A**) Western blot analysis of CARD6 protein expression in lumbar spinal tissues from CARD6-NTG or CARD6-TG mice. (**B**) RT-qPCR and (**C**) western blot analysis of CARD6 expression levels in lumbar spinal tissues from CARD6-NTG or CARD6-TG mice with or without SCI. (**D**) Nissl staining of lumbar spinal cords in the ventral horn of gray matter from mice 3 days after SCI. The number of survived neuron was quantified. Scale bar: 100 μm (black arrows: the normal surviving neurons). (**E**) Western blot analysis was used to calculate GFAP and Iba-1 protein expression levels in the lumbar spinal cord segments. (**F**) Representative images of Caspase-3 and Bcl-2 double staining by IF in dorsal horn of mice. The relative expression of Caspase-3 and Bcl-2 was quantified. Scale bar: 100 μm. (**G**) RT-qPCR analysis of TNF-α, IL-1β and IL-6 mRNA levels in the lumbar spinal cord segments. (**H**, **I**) Western blot analysis of p-IKKα, p-IκBαand p-NF-κB protein expression levels in the lumbar spinal cord segments. Measurements of (**J**) SOD, CAT, (**K**) MDA, H_2_O_2_ and 3-NT in spinal cords of mice at 3 days after SCI. (**L**) RT-qPCR analysis of Nrf2 and Keap1 mRNA expression levels in the lumbar spinal cord segments. (**M**) Western blot analysis for cytosolic Nrf2 and Keap1, and nuclear Nrf2 protein expression levels in the lumbar spinal cord segments. Data represented means ± SEM (n=8 each group). ^*^p < 0.05 and ^**^p < 0.01; ns, no significant difference.

The results above elucidated that CARD6 suppression aggravated SCI progression. To confirm the potential role of CARD6 in regulating SCI, CARD6 TG mice over-expressing CARD6 were created. Western blot analysis indicated that CARD6/TG mice showed higher CARD6 expression than the NTG group (Figure 8A). Then, we found that CRAD6 expression decreased after SCI in CARD6-TG mice compared to CARD6-TG mice from Sham group (Figure 8B and 8C). Nissl staining demonstrated that CARD6 over-expression markedly improved the number of surviving neuron in lumbar spinal cords compared to NTG mice after SCI (Figure 8D). SCI-induced increase of GFAP and Iba-1 was evidently down-regulated in CARD6/TG mice (Figure 8E). IF staining demonstrated that CARD6 over-expression markedly increased Bcl-2 expression and decreased Caspase-3 activation in dorsal horn of mice after SCI, alleviating apoptosis (Figure 8F). Moreover, promoting CARD6 expression significantly reduced the mRNA levels of TNF-α, IL-1β and IL-6 in the lumbar spinal cord segments of mice following SCI, accompanied with apparently reduced phosphorylation of IKKα, IκBα and NF-κB. CARD6 overexpression did not influence basal inflammation under sham conditions (Figure 8G–8I). Sustained CARD6 expression in TG mice clearly improved SOD and CAT activities in spinal cord tissues compared to the NTG mice after SCI (Figure 8J). In contrast, CARD6/TG mice exhibited decreased levels of MDA, H_2_O_2_ and 3-NT in spinal cord samples compared to NTG mice after SCI (Figure 8K). Furthermore, sustained CARD6 expression in SCI mice effectively improved Nrf2 and inhibited Keap1 expression in the lumbar spinal cord segments by RT-qPCR (Figure 8L). Finally, compared to the SCI/NTG mice, CARD6/TG mice after SCI exhibited a significantly reduced expression of Nrf2 and Keap1 in cytosol, whereas nuclear Nrf2 expression was apparently improved (Figure 8M). At the same time, CARD6 over-expression showed no effects on oxidative stress under sham conditions (Figure 8J–8M). Together, the results above elucidated that CARD6 could prevent apoptosis, inflammation and oxidative stress during SCI progression.

**Figure 9:**
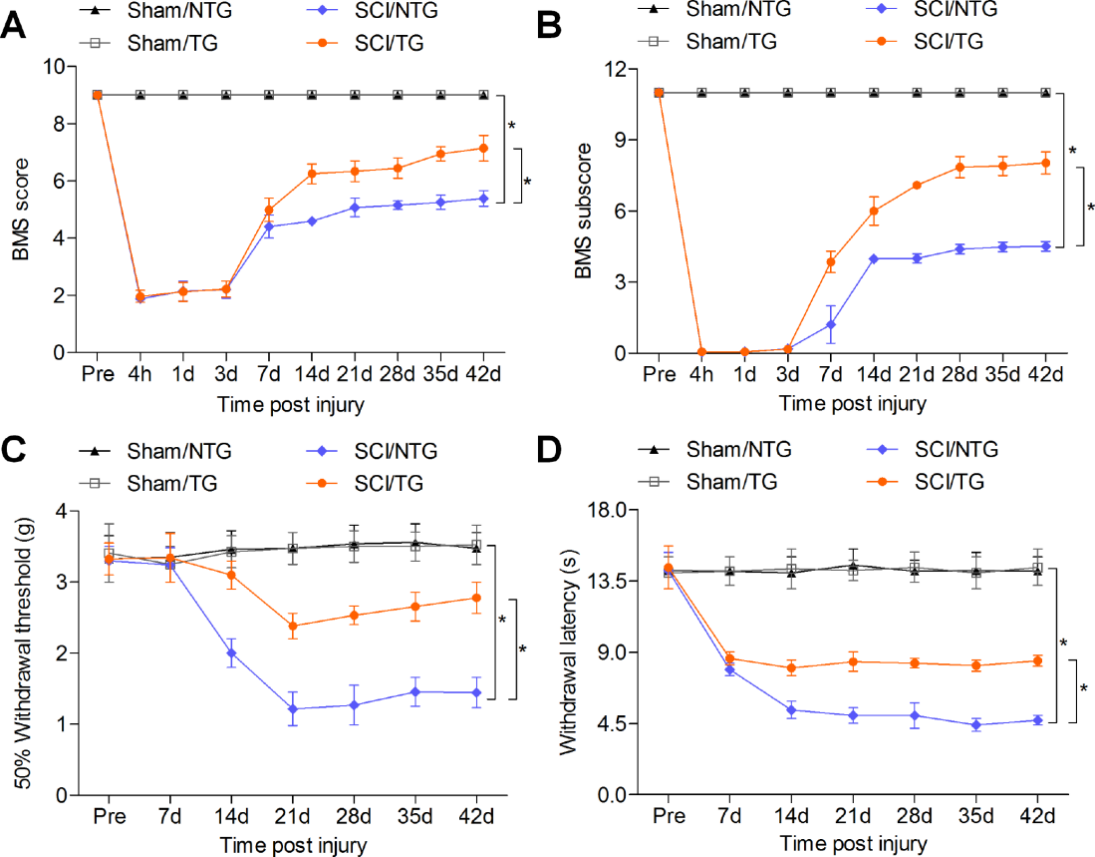
**CARD6 increase improves functional recovery in mice after SCI.** (**A**) The BMS scores and (**B**) BMS subscores were measured in each group of mice. (**C**) The withdrawal threshold was measured to calculate the mechanical hypersensitivity in each group of mice. (**D**) The withdrawal latency was measured to determine the thermal hypersensitivity in each group of mice. Data represented means ± SEM (n=8 each group). ^*^p < 0.05 and ^**^p < 0.01.

Finally, the effects of CARD6 over-expression on functional recovery were verified. As shown in Figure 9A and 9B, SCI-induced decreases in BMS score and BMS subscore were markedly rescued in CARD6/TG mice following SCI. Mice over-expressing CARD6 showed significantly better improvement of the mechanical hypersensitivity compared to the NTG mice after SCI, as evidenced by the increased 50% withdrawal thresholds (Figure 9C). The thermal hypersensitivity in SCI mice was also markedly improved by CARD6 over-expression compared with NTG group, as proved by the up-regulated withdrawal thresholds (Figure 9D). Collectively, these results indicated that sustaining CARD6 expression could improve functional recovery after SCI in mice.

## DISCUSSION

SCI is a fatal condition and typically leads to the loss of sensory and motor functions [1–3]. However, the pathogenesis that contributes to SCI still remains unclear. CARD6 contains a caspase recruitment domain, known as an interaction motif that is found in a wide array of proteins. These proteins are implicated in various processes associated with inflammation and apoptosis [11]. Recently, CARD6 was reported to protect against cardiac hypertrophy in response to pressure overload by regulating mitogen-activated protein kinases (MAPKs) signaling pathway [17]. Furthermore, CARD6-knockout mice exhibited severer fatty livers, which was partly associated with the excessive activation of NF-κB signaling [19]. Similarly, CARD6 efficiently protected against hepatic I/R injury through decreasing the release of inflammatory cytokines, blocking NF-κB signaling and ameliorating the cell death [18]. Nevertheless, the role of CARD6 in SCI progression remains unknown. In the current study, we found that CARD6 expression was gradually down-regulated in spinal cord tissues of mice following SCI, especially at day 3 after SCI. Thus, many protein expressions were examined in samples obtained at 3 days after SCI. However, the motor function recovery was not as quick as the alterations of other gene expression. Therefore, they might be not totally synchronous. Meanwhile, in primary culture astrocytes, microglial cells and mouse microglial BV2 cells, CARD6 expression was significantly reduced by the treatment of TNF-α/IFN-γ or LPS. These results indicated the potential role of CARD6 in regulating SCI development. Then, we found that CARD6-deficient mice exhibited accelerated SCI by promoting neuronal apoptosis, glial activation, inflammation and oxidative stress. However, CARD6 overexpression resulted in the opposite effects, contributing to the functional recovery. The mechanistic studies demonstrated that CARD6 knockdown-enhanced cell death, inflammation and oxidative damage in LPS-stimulated BV2 cells were markedly abrogated by ROS blockage using NAC or PLM. These findings clearly indicated that CARD6 might be a protective modulator in SCI progression.

The occurrence of apoptosis is an important feature of SCI [10, 25, 26]. Increasing studies have suggested that the prevention of apoptosis following SCI could potentially result in spinal cord tissue repair and improve motor function consequently [27, 28]. Here, consistent with previous studies, we found that apoptosis was significantly induced in spinal cord tissues of SCI mice, as evidenced by the significantly reduced expression of Bcl-2, known as a critical anti-apoptotic signal [29], and the enhanced expression of Bax, a key pro-apoptotic molecule [30]. This process subsequently induced mitochondrial membrane permeabilization, leading to the cytoplasm of mitochondrial Cyto-c and the activation of Caspase-3, initiating apoptosis consequently [31]. Recently, CARD6 knockout was suggested to destroy hepatocyte survival mainly through reducing Bcl-2 and enhancing Bax and cleaved Caspase-3 expression [18]. Similarly, here we also found that CARD6-deficient mice showed exacerbated apoptosis in spinal cord samples mainly by promoting Caspase-3 activation, and this was confirmed in LPS-incubated BV2 cells with CARD6 knockdown. In contrast, over-expressing CARD6 exhibited protective against SCI by improving cell survival. Therefore, CARD6 might protect against SCI progression through repressing apoptotic response.

Resolution of inflammation is defective after SCI, which subsequently impairs tissue integrity and remodeling, resulting in functional deficits consequently [5, 32]. Astrocytes and microglia cells are known as primary immune effectors of the spinal cord and facilitate robust activation proximal to the blood-spinal cord barrier in response to SCI [33]. Microglia cell, as the dominant infiltrating cell type, could exacerbate secondary injury acutely through causing axonal dieback, but also promote repair chronically by clearing debris and enhancing remyelination, which is dependent on the microenvironment and their activation state [34, 35]. Targeting immune-regulated secondary injury soon after the primary injury provides a therapeutic avenue to prevent SCI [36]. During SCI development, microglia could be activated partly through the release of pro-inflammatory cytokines, such as TNF-α, IL-1β and IL-6 [37, 38]. In the present study, we found that the expression of TNF-α, IL-1β and IL-6 was significantly induced in spinal cord samples of mice after SCI, which was notably accelerated by CARD6 knockout, further contributing to the spinal cord damage. These pro-inflammatory cytokines are mainly meditated by the transcription factor NF-κB, which induces a self-perpetuating process of progressive neuroinflammation [39]. CARD6 was suggested to show a positive role in NF-κB activation [12, 15]. However, there was also a study indicating that CARD6 suppressed activation of NF-κB by NOD1 or RIPK2 but did not interfere with NF-κB activation [16]. Thus, there is a conflicting role of CARD6 in NF-κB activation. Recently, CARD6-knockout mice showed significantly promoted hepatic I/R-induced NF-κB activation, whereas CARD6 over-expression abolished the activation of NF-κB, contributing to the decreases in the release of pro-inflammatory cytokines [18]. In addition, CARD6-deficient mice exhibited accelerated fatty liver, which was also highly associated with the increase of NF-κB activation, while being alleviated in mice over-expressing CARD6 [19]. In accordance with these findings, we also observed a negative regulation by CARD6 on NF-κB signaling, as well as on the cytokine production, providing unambiguous evidence that CARD6 played a pivotal role in the pathogenesis of the classical NF-κB-associated inflammatory response. Since CARD6 played a critical role in regulating inflammation following SCI, and the suppressive role of LPS in meditating CARD6 expression, we supposed that repressing inflammatory response might affect CARD6 expression after SCI. As for this, further studies are still warranted in future to comprehensively reveal the underlying molecular mechanisms.

SCI leads to production of massive amounts of ROS that could directly damage the main cellular constituents [6–9, 40]. The central nervous system is extremely sensitive to oxidative stress because of delicate lipid layers of its cell membranes and reduced levels of antioxidant enzymes [41]. Nrf2, as the transcription factor, is involved in the adaptation and survival of cells under stress conditions through modulation of mitochondrial function by employing multiple networks of cytoprotective proteins [42, 43]. Under homeostatic conditions, Nrf2 influences mitochondrial membrane potential, fatty acid oxidation, the availability of substrates for respiration, as well as ATP synthesis [44]. But under stress conditions, Nrf2 detaches from Keap1 and translocates to the nucleus, and counteracts the promotion of ROS production through transcriptional increase of antioxidant proteins, including SOD, CAT, HO1, NQO1, GCLM and GCLC, which subsequently modulate oxidative stress, apoptosis and inflammation in diverse neurological disorders [45, 46]. Previous studies have documented that after SCI, oxidative stress markers specific to lipid and protein oxidation, namely 4-HNE and 3-NT, all up-regulate in the injured tissue homogenates [47, 48]. Here, our study further confirmed that SCI mice exhibited significant oxidative stress, as evidenced by the reduced activities or expression levels of SOD, CAT, GSH, HO1, NQO1, GCLM and GCLC; however, MDA, 4-HNE and 3-NT were markedly up-regulated in spinal cord tissues from mice after SCI. This process was largely associated with the down-regulation of Nrf2 activation that translocated into nuclear to produce anti-oxidants. Intriguingly, for the first time we revealed that CARD6 knockout evidently accelerated SCI-induced oxidative stress by Nrf2 blockage in mice, which was verified in LPS-incubated BV2 cells with CARD6 knockdown. Notably, promoting CARD6 expression in mice showed suppressive effects on SCI-induced oxidative stress by improving Nrf2 activation. Accumulating evidences have indicated that preventing ROS generation could suppress astrocytic hyperactivation through blocking NF-κB signaling [49, 50]. The activation of BV2 microglia cells could also be repressed by reducing ROS accumulation, which was associated with the inactivation of NF-κB signaling [51]. Moreover, stresses-induced apoptosis was also related to ROS production in multiple cell types, including glial cells [52, 53]. For instance, spinal cord ischemia results in metabolic disorders of oxygen and produces excessive ROS, leading to mitochondrial membrane permeabi-lization, release to the cytoplasm of mitochondrial Cyto-c, as well as Caspase-3 activation, consequently enhancing apoptosis and spinal cord damage [54]. In the present study, we significantly found that reducing ROS production by NAC or PLM, known as critical inhibitors of ROS, could abrogate CARD6 knockdown-promoted apoptosis, inflammatory response and oxidative stress-regulated by Nrf2 in BV2 cells stimulated by LPS. These results indicated that CARD6-regulated cell death, inflammation and oxidative damage in SCI were largely through its blockage to ROS production. Presently, the detailed molecular mechanism by which CARD6 improves Nrf2 activation is unclear and should be further investigated in future studies.

In conclusion, our study provided the first evidence, to our knowledge, that CARD6 was an important protective factor against SCI progression by preventing apoptosis, inflammation and oxidative stress, which might be associated with ROS production regulated by Nrf2 signaling pathway. These findings established a working model encompassing the function of CARD6 in SCI and demonstrated a potential drug target for the treatment of these common diseases.

## MATERIALS AND METHODS

### Animals and treatments

All procedures involving animals were approved by the institutional animal care and use committee of the Affiliated Hospital of Southwest Medical University (Sichuan, China), and were reported in accordance with the ARRIVE (Animals in Research: Reporting *in vivo* Experiments) guidelines. All efforts were performed to minimize the suffering and reduce the number of animals used. The wild type (CARD6^+/+^) C57BL/6 mice were purchased from the Laboratory Animal Centre in West-China Center of Medical Sciences, Sichuan University (Sichuan). The wild type male mice were 10 weeks old and weighed 24-26 g at study initiation. The CARD6 knockout (CARD6^-/-^) and CARD6 transgenic mice (TG) C57BL/6 mice were created and purchased from Cyagen Biotechnologies (Guangzhou, China). To generate CARD6-TG mice, we cloned full-length murine CARD6 cDNA downstream of the glia-specific promoter platelet-derived growth factor. This construct drove the preferential expression of CARD6 in glial cells. TG mice were produced through microinjecting the construct into fertilized embryos (C57BL/6 background), and four independent transgenic lines were established. Only 10-11-week-old (24-26 g) males were used. All animals were housed in a specific pathogen-free (SPF) laboratory animal room and given access to a 12 h light-dark cycle in a 18-22°C facility with 40-60% humidity and free access to water and food ad libitum. The mice were anesthetized ketamine (100 mg/kg, i.p.) and xylazine (15 mg/kg, i.p.). During surgery, the rectal temperature was maintained at 37°C by a heating pad. After laminectomy at T10, SCI was performed using a modified New York University Impactor as previously described [55]. A 10 g rod (tip diameter: 1.5 mm) was then dropped from 3 mm onto the T10 segment. The SCI was moderate injury and neural tissue damage was mainly occurred in the dorsal column of the spinal cord. After SCI, bladders were expressed twice a day until the bladder reflex was re-established. Sham control group of mice receiving laminectomy without SCI were prepared.

As for LPS-treated spinal cord injury, the wild type mice (age, 8-10 weeks; weight, 23-25 g) were divided into two groups: i) Control (Con) group, which received the same volume of 0.1% DMSO vehicle diluted in saline (0.1 ml/10 g body weight); ii) LPS group, which received an intraperitoneal (i.p.) injection of LPS (0.25, 0.5, or 1 mg/kg) for 5 consecutive days. Five mice per cage were housed in transparent plastic cages in controlled conditions at 18-22°C with 40-60% humidity and a 12 h light/dark cycle. All mice were allowed ad libitum access to water and food. After LPS injection, all mice were sacrificed. The spinal cord segments (dorsal part of L4-L5) were collected for RT-qPCR and western blot analysis.

### Cells isolation and culture

Mouse BV2 microglia cells were purchased from China Center for Type Culture Collection (CCTCC, Wuhan, China). BV2 cells were cultured in DMEM high-glucose complete medium (Gibco, USA) supplemented with 10% fetal bovine serum (FBS, Gibco), 100 U/mL penicillin and 100 μg/mL streptomycin, and then were incubated in an incubator at 37 °C with a volume fraction of CO_2_ of 5%. Oligodendrocyte precursor cells (OPCs) were generated from primary mixed glial cultures as described previously [56]. Purified OPCs were planted onto poly-L-lysine (PLL)-coated 6-well plates and cultured in an oligodendrocyte growth medium containing basal chemically defined medium (BDM) (DMEM/F12 supplemented with 4 mM L-glutamine, 50 μg/ml streptomycin, 50 U/ml penicillin, 0.1% bovine serum albumin, 1 mM sodium pyruvate, 50 μg/ml apo-transferrin, 5 μg/ml insulin, 30 nM sodium selenite, 10 nM biotin, and 10 nM hydrocortisone) supplemented with 10 ng/ml PDGF-AA and 10 ng/ml bFGF to promote cell growth. OPCs were cultured in the above growth medium for 2-3 days and passaged with DMEM/F12containing 0.01 % EDTA, 0.2 mg/ml DNase, and 5 μg/ml insulin. Primary microglia cells were obtained from the cerebral hemispheres of newborn mouse brains as previously described [57]. Briefly, mixed glial cells were plated onto PLL-coated culture flasks. Two weeks later, microglia were shaken off at 200 rpm for 30 min. A highly enriched microglial suspension was collected and filtered using a 41 μm cell strainer. After centrifugation, cells were resuspended in DMEM supplemented with 10% FBS. The purity of obtained cells >95% was harvested according to Iba-1 staining before treatments. Primary astrocytes were cultured from the cerebral cortex of newborn mouse brains as previously described [58]. Briefly, the hemispheres were carefully dissected out, the meninges of hypothalamus was cautiously separated, and then minced by sterile surgical scissors and dissociated with 0.25% trypsin/1 mM EDTA. The fragments were then washed using cold D-Hank’s buffer (Gibco) and the meninges were gingerly removed. The re-suspended cells were harvested and planted in uncoated culture flasks containing medium (DMEM/F12 supplemented with 10% FBS, 1×10^5^ U/L streptomycin sulfate, pH 7.2) with a concentration of 1×10^6^/ml at 37°C, 5% CO2. Confluent cultures were passaged by trypsinization, and astrocytes were isolated through shaking. The 3 passages cells were subjected to further studies. TNF-α, IFN-γ, lipopolysaccharide (LPS), N-acetylcysteine (NAC) and piperlongumine (PLM) were purchased from Sigma Aldrich (USA).

### Cell transfection

CARD6 short interference RNA (siCARD6) and the negative control (siCon) were purchased from Generay Biotech (Shanghai, China) were transfected to cells for 24 h using Lipofectamine 3000 (Invitrogen, USA) according to the manufacturer’s protocol.

### Tissue preparation and histological analysis

The spinal cord lumbar segments containing the injured region were fixed in 4% paraformaldehyde overnight. The segments were then transferred into 30% sucrose in 4% paraformaldehyde until reaching the bottom. Serial 6 μm transverse frozen sections were cut with a cryostat (Leica CM 1850, Leica Microsystems, Swiss). Adjacent sections were subjected to H&E staining. Apoptotic cells were measured using TUNEL staining with an In situ Cell Death Detection kit (Roche, Shanghai, China) according to the manufacturer’s protocols. Then, nuclei were labeled with DAPI (Sigma Aldrich) and analyzed by an independent observer with a fluorescent microscope. As for Nissl staining, the sections were incubated in 0.1% Cresyl violet Nissl staining solution following the manufacturer’s instructions (Beyotime, Shanghai, China). The quantity of neurons of five sections from each mouse was analyzed under a light microscope by two pathologists blinded to the treatments and outcomes.

### Immunohistochemical (IHC) staining

Tissue sections were blocked for 1 h in 5% bovine serum albumin (BSA, Sigma Aldrich) and incubated with primary antibodies (Supplementary Table 1) overnight at 4°C. Sections were then washed in PBS and incubated with a secondary antibody conjugated to horseradish peroxidase (HRP) secondary antibodies (1:500, Invitrogen) for 1 h at room temperature. Then, sections were washed with PBS and visualized by chromogen DAB (Sigma Aldrich) reaction. Finally, the spinal cord sections were dehydrated in ethanol, cleared in xylene, mounted with Permount (Thermo Fisher Scientific, Rockford, USA), and analyzed with a light microscopy.

### Immunofluorescence (IF) staining

After being transferred to 30% sucrose solutions, the spinal cords of mice were cut into 6 μm sections using a cryostat microtome. Sections were then blocked for 1 h in 10% BSA and incubated overnight at 4°C with primary antibodies (Supplementary Table 1). Sections were then washed with PBS and incubated for 1 h at room temperature with corresponding secondary antibodies conjugated to Alexa Fluor 488 or 594 (1:500, Invitrogen). Sections were then washed in PBS, incubated with DAPI (Sigma Aldrich) in the dark. Representative images were captured using a fluorescent microscope (Microscope Axio Imager. A2, Carl Zeiss, Germany).

### Western blotting

The nuclear and cytoplasmic fractions were prepared using a Nuclear Extraction Kit (Abcam, Cambridge, USA). The mitochondrial and cytoplasmic fractions were isolated using a Mitochondria/Cytosol Fractionation Kit (BioVision Inc., Mountain View, USA). For whole cell lysates, the spinal cord tissues containing the injured region and treated cells were homogenized using lysis buffer (NanJing KeyGen Biotech Co.,Ltd., Nanjing, China). The protein concentrations of each sample were measured using a BCA protein assay kit (Beyotime). An equal amount of total protein (40 μg) was separated on sodium dedecyl sulfate-polyacrylamide gels (SDS-PAGE), and transferred onto polyvinylidene difluoride membranes (PVDF, Millipore, Billerica, USA). The membranes were then blocked for 1 h in 5% skim milk solution, and incubated with specific primary antibodies (Supplementary Table 1) at 4°C overnight. The blots were then incubated with the corresponding conjugated horseradish peroxidase (HRP) secondary antibodies (1:8000; Santa Cruz, USA). Immunoreactive proteins were detected using an enhanced chemiluminescence Western blotting detection system (Thermo Fisher Scientific). The relative density of the protein bands was quantified using ImageJ software (NIH, Bethesda, USA). Nuclear samples were normalized against Lamin B, mitochondrial samples were normalized to cytochrome c oxidase IV (COX IV), cytoplasmic and whole cell extracts were normalized against GAPDH.

### Quantitative real-time PCR (RT-qPCR)

Indicated gene expression was measured using RT-qPCR as previously described [59]. Briefly, the total RNA in spinal cord tissues containing the injured region or cells was extracted using Trizol reagent (Takara, Tokyo, Japan) according to the manufacturer’s instruction. cDNA was then synthesized from an equivalent amount of RNA using the Super M-MLV reverse transcriptase (Biotech, Beijing, China). Next, qPCR was carried out using the 2xPower Taq PCR MasterMix (Biotech) and SYBR Green PCR Master Mix (Takara). GAPDH was used as an internal control. Primers used in the study were exhibited in Supplementary Table 2. All reactions were performed in triplicate.

### Measurement of cytokine levels

The contents of TNF-α (#MTA00B), IL-1β (#MLB00C) and IL-6 (#M6000B) in medium were quantified with commercially available ELISA kits according to the manufacturer’s protocols (R&D Systems, Minneapolis, USA).

### Assessment of ROS production

DCFDA/H2DCFDA-Cellular ROS Assay Kit (ab113851, Abcam) was used to determine the ROS production in cells according to the manufacturer’s protocols.

### Biochemical detection

Spinal cord tissue samples were collected and lysed using RIPA buffer (NanJing KeyGen Biotech Co.,Ltd.). Then, the activities or levels of SOD (#A001-3-2), CAT (#A007-1-1), GSH (#A006-2-1), MDA (#A003-1-2) and H_2_O_2_ (#A064-1-1) were measured using corresponding commercial kits (Jiancheng Bioengineering Institute, Nanjing, China) according to the manufacturer’s instructions. The contents of 3-NT (#ab116699, Abcam) in spinal cord tissues were measured using commercial kit according to the manufacturer’s protocols.

### Locomotor function

To calculate the functional consequences of SCI, locomotor rating tests were performed for 42 days after SCI using the Basso Mouse Scale (BMS) score and the BMS subscore [60]. Before surgery, animals were individually placed in a molded plastic open field for 4 min to confirm that all subjects consistently obtained the maximum score. Then, the experimental animals were placed in the open field, and well-trained investigators scored them on the BMS in a blinded manner. The BMS scores were analyzed for the left and right hind limbs. The averaged value was obtained.

### Hypersensitivity analysis

The mechanical hypersensitivity of the plantar hindpaws was measured according to the von Frey method weekly until 42 days after SCI [61]. The cutaneous sensitivity to innocuous mechanical stimulation of both hindpaws was calculated by the up-down method with eight specific calibrated the von Frey filaments [62]. The series of responses to the filaments was converted into a 50% withdrawal threshold, which was measured in grams [63]. The thermal hypersensitivity of the plantar hindpaws was evaluated as previously described [64]. Briefly, animals were placed allodially in individual clear plastic compartments on an elevated glass floor. The Plantar Test Apparatus (model 37370, Ugo Basile, Comerio, Italy) was used through the glass floor to the middle of the plantar surface of the hindpaws. When the mouse lifted its paw, a photocell automatically stopped the heat source and the timer. The latency to withdrawal from the heat source was recorded. In order to prevent tissue injury, a cut-off of 20 s was applied. The average of three trials/paw was analyzed to indicate the final withdrawal latency.

### BDA tracing

Biotinylated dextran amine (BDA) (Invitrogen, USA) was injected to the right sensorimotor cortex 3 days following the injury. The tracer was then injected at 4 sites (0.4 μl per site over a period of 5 min, plus 3 min of the glass capillary in place to avert spillover), coordinates (from bregma) were 1.0 mm lateral, 0.5 mm deep, and +0.5, -0.2, -0.7, and -1 mm. The mice were sacrificed two weeks after injection.

### Statistical analysis

All data were presented as the means ± SEM by statistical analysis of Graph Pad Prism software (Graph Pad Software, La Jolla, CA, USA). BMS and BMS subscore data were analyzed by two blinded investigators using repeated measures ANOVA with Bonferroni’s multiple comparisons test. All other analysis were performed using unpaired t test or one-way ANOVA as appropriate. The P value less than 0.05 was considered significant.

## Supplementary Material

Supplementary Figure 1

Supplementary Tables
