# Supplementary material for: Targeting CARD6 attenuates spinal cord injury (SCI) in mice through inhibiting apoptosis, inflammation and oxidative stress associated ROS production
Source: Aging (Albany NY). 2019 Dec 16;11(24):12213–35. doi: 10.18632/aging.102561 (PMC6949089; doi:10.18632/aging.102561)
Supplement: Supplementary Figure 1 [file aging-11-102561-s001..pdf]

SUPPLEMENTARY FIGURE

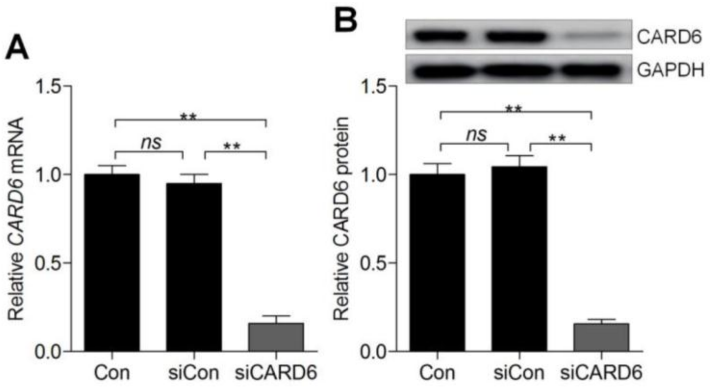

Supplementary Figure 1. (**A**) RT-qPCR and (**B**) western blot analysis of CARD6 in BV2 cells transfected with siCon or siCARD6 for 24 h. Data represented means ± SEM(n=6 each group). $^{**}p < 0.01$; ns, no significant difference.
