# Supplementary material for: Targeting CARD6 attenuates spinal cord injury (SCI) in mice through inhibiting apoptosis, inflammation and oxidative stress associated ROS production
Source: Aging (Albany NY). 2019 Dec 16;11(24):12213–35. doi: 10.18632/aging.102561 (PMC6949089; doi:10.18632/aging.102561)
Supplement: Supplementary Tables [file aging-11-102561-s002..pdf]

SUPPLEMENTARY TABLES

Supplementary Table 1. Primary antibodies for western blot (WB), IHC or IF analysis.

Antibodies	Catalog	Dilutions			Vendor
		WB	IHC	IF	
CARD6	Ab151560	1:1000	N/A	N/A	Abcam
GAPDH	Ab8245	1:1000	N/A	N/A	Abcam
Phospho-IKKα	Ab59195	1:1000	N/A	N/A	Abcam
IKKα	Ab32041	1:1000	N/A	N/A	Abcam
Phospho-NF-κB	Ab86299	1:1000	N/A	N/A	Abcam
NF-κB	#436700	1:1000	N/A	1:150	Invitrogen
Phospho-IκBα	#PA1-85928	1:1000	N/A	1:150	Invitrogen
IκBα	Ab32518	1:1000	N/A	N/A	Abcam
GFAP	Ab7260	1:1000	N/A	1:150	Abcam
Iba1	Ab153696	1:1000	N/A	1:150	Abcam
Cyto-c	Ab13575	1:1000	N/A	1:150	Abcam
Nrf-2	Ab62352	1:1000	N/A	1:100	Abcam
COX IV	Ab33985	1:1000	N/A	N/A	Abcam
Keap-1	Ab150654	1:1000	N/A	N/A	Abcam
Bcl-2	#MA5-11757	1:1000	N/A	1:100	Invitrogen
Bax	Ab32503	1:1000	N/A	N/A	Abcam
Lamin B	Ab133741	1:1000	N/A	N/A	Abcam
Caspase-3	Ab49822	1:1000	N/A	1:150	Abcam
TNF-α	Ab6671	N/A	1:200	N/A	Abcam
IL-1β	Ab9722	N/A	1:100	N/A	Abcam
5HT	QY0908R	N/A	N/A	1:100	QIYBO
CD68	Ab125212	N/A	N/A	1:150	Abcam
F4/80	Ab6640	N/A	N/A	1:150	Abcam
NeuN	Ab177487	N/A	N/A	1:100	Abcam
4-HNE	#MA5-27570	N/A	N/A	1:100	Invitrogen

Supplementary Table 2. Primer sequences used in the present study.

Primers	Forward Sequence (5'-3')	Reverse Sequence (5'-3')
CARD6	AGCACTCTGTATCTACATGAC	ATGATGCCATTATAAGACCGA
SOD1	CTTACCTAATAGGCCTAACGA	AACAGCACACCACAACATCC
SOD2	GTCTGGCTCTACTGGAATGTC	CACCATGGCAGACACTGTAGA
Nrf2	TGAACACTCTGGAGATGACA	GGACTCTGGTCTTTGTGTTC
NQO1	GGTTTACAGCATTGGCCACACT	AACAGGCTGCTTGGAGCAAA
GCLM	CCACCTCAGGGTCACAAGGT	TCACGCTCCTTGATATGCAC
TNF-α	ACCTGGCCTCTCTACCTTGT	CCCGTAGGGCGATTACAGTC
IL-6	CAACGATGATGCACTTGCAGA	TCTCTCTGAAGGACTCTGGCT
IL-1β	TCGCTCAGGGTCACAAGAAA	CATCAGAGGCAAGGAGGAAAAC
GCLC	ACATTTCTTGGACCCCACGA	GGTTGGTCTGTCACACTTGC
HO1	TGAACACTCTGGAGATGACA	GGACTCTGGTCTTTGTGTTC
Keap1	CCTCTGCCTTCACTACACTGAGATT	TGTGGAAGCCTTCCTGGATG
GAPDH	GAACAGTGTGGTCGGTGAGG	TTACCGGGCCCAAGTGTAGC
